# Recovery from disorders of consciousness: Lesions and GABAergic modulation in a biologically inspired spiking neural network

**DOI:** 10.1073/pnas.2530522123

**Published:** 2026-09-22

**Authors:** Francesco Achilli, Antoine Cautru, Jean-Pierre Changeux, Guillaume Dumas

**Affiliations:** ^a^ Department of Biomedical and Clinical Sciences “Luigi Sacco”, University of Milan, Milan 20157, Italy; ^b^ Research Center of the Sainte-Justine Mother and Child University Hospital Center, Department of Psychiatry and Addictology, University of Montreal, Montreal H3T 1C5, QC, Canada; ^c^ Neuroscience Department, Institut Pasteur, Collège de France, Paris F-75005, France; ^d^ Mila—Québec AI Institute, University of Montreal, Montreal H2S 3H1, QC, Canada

**Keywords:** disorders of consciousness, global neuronal workspace, spiking neural network, GABAergic modulation, excitation–inhibition balance

## Abstract

The effects of positive allosteric modulators (PAMs) of the GABA_A_ receptor in disorders of consciousness (DoC)—such as the hypnotic zolpidem in coma—are frequently reported but remain poorly understood. Although several hypotheses have been proposed to explain their paradoxical effect, biologically plausible computational models that test why only a subset of patients with DoC respond to inhibitory-enhancing medications may clarify this phenomenon and guide therapeutic strategies. We extended a spiking neural network model to mechanistically investigate inhibitory neuron enhancement in DoC. The model featured biologically inspired elements, including a ratio of 80:20 excitatory-to-inhibitory neurons and 12 Hz of spontaneous intrinsic activity. Neurons were spatially organized, and the network performed a trace conditioning task to investigate conscious access. We implemented localized and diffuse lesions affecting excitatory and inhibitory neuron populations, individually and in combination. The positive allosteric modulation of GABA_A_ receptors was simulated by increasing inhibitory synaptic weights. Experiments revealed distinct recovery patterns depending on damage type and lesion distribution. Spontaneous recovery was more impaired by excitatory than inhibitory lesions, with local lesions generally affecting performance more than diffuse ones. Inhibitory postsynaptic potentiation produced dose-dependent recovery after inhibitory lesions, with therapeutic windows varying by lesion type. Our findings suggest that the paradoxical effect of GABA_A_ receptor PAMs arises from the restoration of excitatory–inhibitory balance when inhibitory networks are moderately disrupted. This computational framework offers a testable account in which insufficient inhibitory regulation represents a common pathway underlying DoC cases responsive to GABA_A_ receptor PAMs across diverse etiologies.

## Significance

This study presents a computational framework to mechanistically simulate neuronal lesions and interventions for the treatment of disorders of consciousness (DoC). Through a neural network with biologically plausible features, we modeled different lesion types and varying degrees of GABAergic positive modulation, showing how zolpidem’s therapeutic effects may depend primarily on the pattern and severity of inhibitory network damage. Our findings highlight the critical role of excitatory–inhibitory neuron balance in access to consciousness and point toward a pathway for precision medicine in DoC.

Disorders of consciousness (DoC) are characterized by alterations in arousal and awareness (1) and are due to brain injuries of ischemic, traumatic, metabolic, toxic, or autoimmune origin (2).

Despite the varied etiology of neurological damage, the mechanism underlying its clinical presentation is generally attributed to disruptions in neural connectivity (3). Several mechanisms have been proposed to explain DoC unfolding, mainly focusing on disruptions of corticostriatal and thalamocortical circuits (4) and deviations in large-scale network dynamics (5).

Because a reduction in excitatory tone was traditionally considered the sole mechanism underlying DoC, the beneficial effects of zolpidem—a selective α1 subunit GABA_A_ receptor positive allosteric modulator (PAM) (6)—commonly prescribed for insomnia (7), were initially described as a neuropharmacological paradox (8). It is now estimated that only 5 to 7% of patients with DoC respond to zolpidem (9). Remarkably, other GABAergic agents have demonstrated similar paradoxical effects: Propofol, a nonselective GABA_A_ PAM (10), is being studied for occasional paradoxical brain responses and potential therapeutic effect in DoC (11); baclofen, a selective GABA_B_ receptor agonist (12), mediating a slower and more modulatory form of inhibition (13), has shown potential in neurorehabilitation (14, 15). To explain why only a subset of patients respond to such GABAergic compounds, two main models have been proposed: the mesocircuit model (16), which highlights loss of striatal inhibition of the globus pallidus (GP), and the hypothesis of a supercritical state (17), pointing to an imbalance toward hyperintegration of neuronal signal potentially normalized by these medications.

When networks deviate from stability, neural systems carry out adaptive strategies: Biological models suggest that redundancy and plasticity help preserve function after perturbations (18). Accordingly, synaptic epigenesis (19–21) accounts for the brain’s capacity to adapt and reorganize when challenged with pathological insults (22, 23), environmental demands (24), or, when intrinsic readaptation is insufficient, therapeutic interventions (25). Hypothetically, GABAergic medications may redress the excitatory–inhibitory neuron balance (E:I ratio), whose disruption undermines electrophysiological and metabolic homeostasis (26).

Thanks to the latest advances in computational neuroscience (27), formal models can be used to investigate mechanistic hypotheses, allowing for the simulation and manipulation of complex neural interactions in silico, which are otherwise difficult to observe directly in vivo (28). Spiking neural networks (SNNs) offer biologically plausible platforms for simulating interactions between excitatory and inhibitory neurons (29). Although SNNs typically underperform traditional artificial neural networks (ANNs) in standard computational tasks (30), they can incorporate temporal dynamics and emulate neuronal plasticity (31). A recent model developed by Volzhenin et al. (21) demonstrated that the development and function of the Global Neuronal Workspace (GNW) depend on biologically grounded parameters, such as the E:I ratio and spontaneous intrinsic activity. The model’s sensitivity to E:I ratio may offer possible explanations for how shifts from physiological homeostasis, and the action of GABA_A_ receptor medications, may affect compromised networks.

In this study, we extended the model developed by Volzhenin et al. (21) to investigate the consequences of GABA_A_ receptor modulation in DoC. Our simulations scale the network to a larger number of neurons and incorporate spatial localization to reflect structural network architecture. We further introduce targeted lesions and simulate inhibitory modulation to examine recovery trajectories. We hypothesize that the efficacy of GABAergic PAMs depends on pattern and severity of inhibitory network damage and that reinstatement of inhibitory drive is the common pathway underlying responsive cases.

## Results

**Fig. 1.**
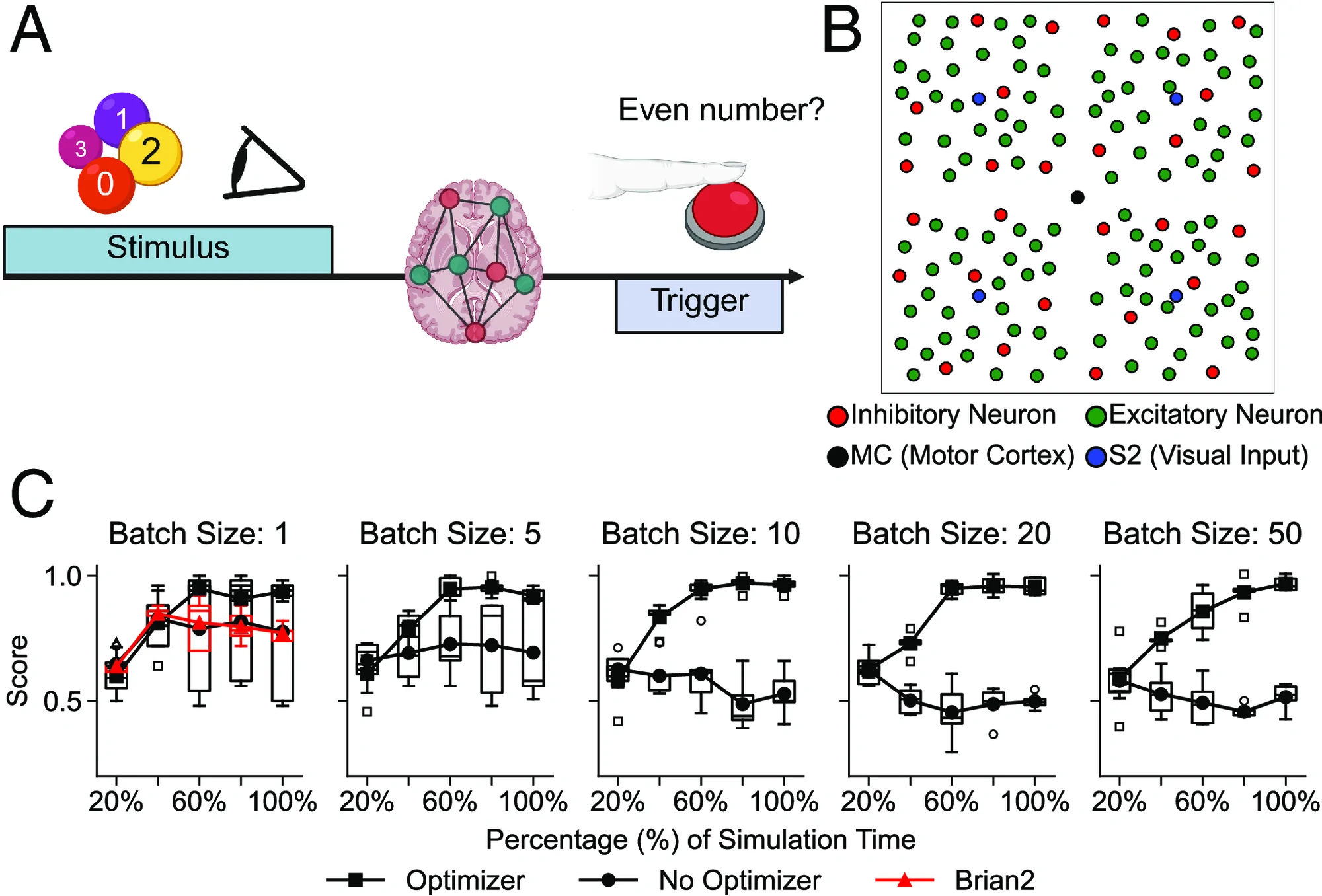
Task description, neuronal workspace and columnar architecture, and model improvements. (*A*) Task description. Each episode (total duration: 1 s) begins with a visual stimulus represented by a random digit between 0 and 3. To investigate conscious access, a delay between stimulus presentation and the trigger is introduced. During this interval, sustained network activation is expected, maintained by excitatory–inhibitory neuron balance. Finally, the model is prompted to indicate whether the digit was an even number. Correct responses score one point; incorrect responses zero. (*B*) Neuronal Workspace and columnar architecture. While S2 (Visual Input) and MC (Motor Cortex) neurons represent sensory integration and motor function, the inhibitory and excitatory neurons simulate the cognitive and conscious level. (*C*) Model improvements. Task performance (score, 0 to 1; 0.5 = chance) across five consecutive time windows, for batch sizes 1 to 50. Lines show the mean across n = 5 independent trials; box plots show the median (center line), interquartile range (box), and 1.5 × IQR (whiskers); outliers shown as open markers. The PyTorch implementation with connectivity normalization (Optimizer) consistently outperforms the version without optimization (No Optimizer) across all batch sizes and exceeds the original Brian2 model (shown for batch size 1) at matched simulated time.

After implementing our SNN within a clock-driven framework (32), inspired by the machine-learning oriented BindsNET library (33), we replicated the trace conditioning task (Fig. 1*A*) from Volzhenin et al. (21), featuring sensorimotor and conscious levels (Fig. 1*B*) to investigate conscious access [the ability to retain and utilize information (34)]. The reference implementation computed Spike-Timing-Dependent Plasticity (STDP) through event-driven synaptic updates in Brian2 (35), while our PyTorch-based model updated plasticity clock-driven at every timestep. After replicating the original dynamics, introducing connectivity normalization (*SI Appendix*, Table S1) raised final performance (score 0.94 vs. 0.77) and reproducibility across trials (coefficient of variation 0.029 vs. 0.043) at matched simulated time (Fig. 1*C*). Computing STDP synchronously enabled batching: Identical networks ran in parallel with shared synaptic weights but independent inputs and membrane potentials, so their plasticity updates were averaged across the batch and normalized to maintain stability (*SI Appendix*). Batching reduced the simulated time required to reach a performance threshold (a score of 0.8 required 100 s of simulated time without batching, but only 3 s at a batch size of 50), balancing computational efficiency with the model’s biological foundations.

### Network Size and Spatialization.

We first examined if network size influenced stability. The SNN maintained learning and optimal performance with up to 400 neurons at the cognitive and conscious level while preserving the 80:20 E:I ratio.

After introducing neuron’s spatialization, we tested network sizes following two different approaches: i) increasing the number of neurons within each column and ii) increasing the number of columns. With a fixed column size of 30 neurons, the network remained stable with up to 36 columns, corresponding to a total of 1,080 neurons. When increasing the number of neurons per column, in a network of four columns, stability and performance were maintained up to a column size of 50 neurons. Subsequent experiments were performed on a network of four columns and column size of 36 neurons. To compare and validate spontaneous recovery patterns after lesions, further lesion experiments were executed on a network of four columns and column size of 50 neurons.

### Lesion Experiments.

**Fig. 2.**
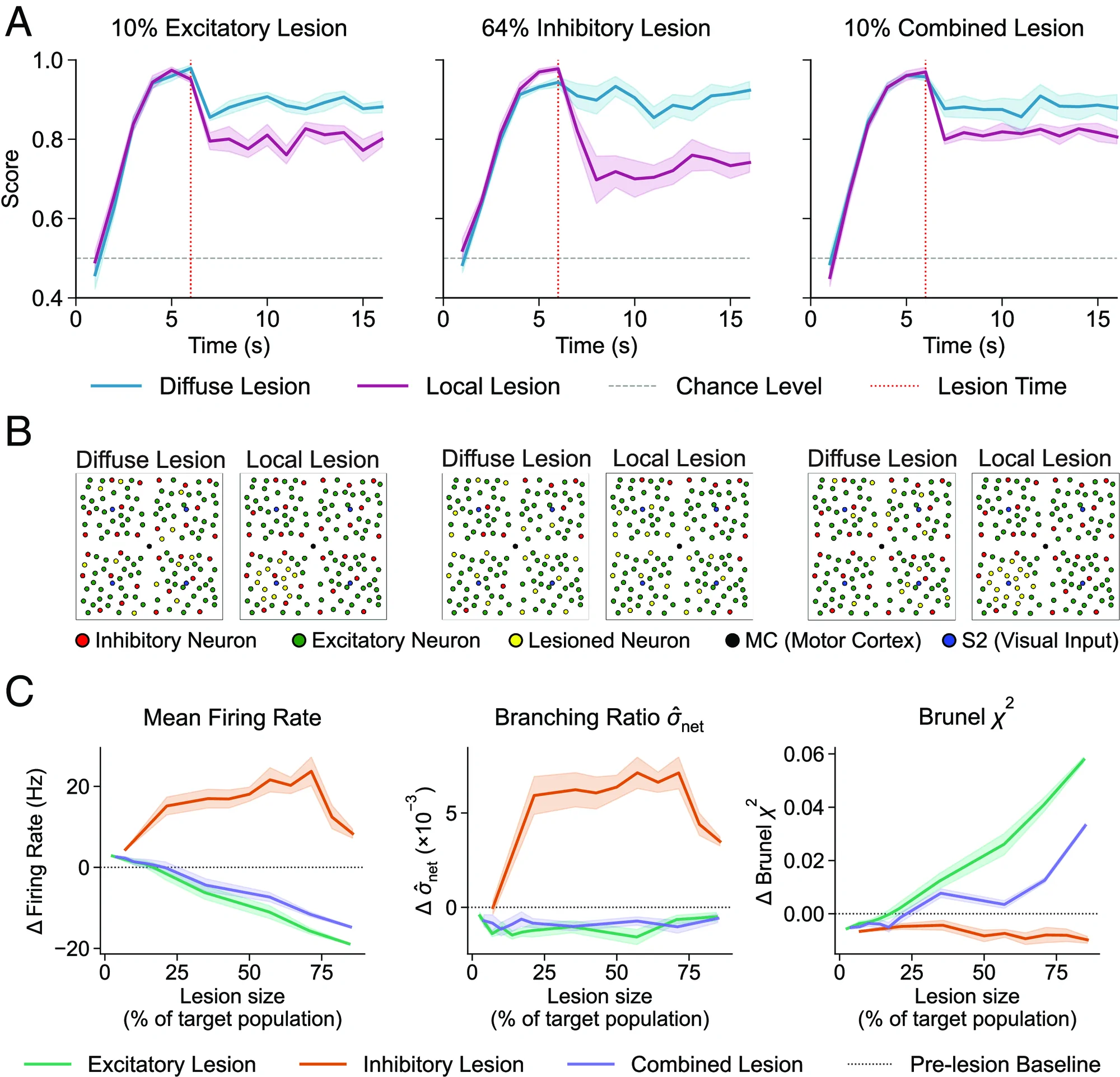
Spontaneous recovery patterns, lesion distributions, and network dynamics. (*A*) Spontaneous recovery patterns. Score (0 to 1; 0.5 = chance, dashed line) over time after diffuse and local lesions. Solid lines show the mean and shaded bands the ±SEM across n = 10 independent trials per condition. Small excitatory or combined lesions allow partial recovery, with local lesions hindering recovery more than diffuse ones. This lesion-type effect is also observed for inhibitory lesions. However, for inhibitory lesions, a greater damage is required to prevent recovery. (*B*) Lesion distributions. Representative damage pattern for each condition shown in panel *A* (*Left* to *Right*: 10% excitatory, 64% inhibitory, 10% combined lesions). Diffuse lesions spread the damage across the network, favoring better spontaneous recovery than localized lesions. (*C*) Dynamical characterization across lesion sizes. Changes, relative to prelesion baseline, in neuronal firing rate (*Left*), branching ratio ${\hat{\sigma }}_{net}$ (*Middle*), and Brunel synchrony index χ^2^ (*Right*) plotted against lesion size (% of target population). Solid lines show the mean and shaded bands the ±SEM across n = 10 independent trials per lesion size. Inhibitory lesions cause a nonmonotonic shift in firing rate and branching ratio while reducing synchrony; excitatory and combined lesions induce a steady reduction in network activity and a more synchronized regime, with a sustained reduction in the branching ratio. This shows dissociable dynamical signatures after distinct circuit disruption.

Local lesions, with deactivation of neural activity in a confined region, were designed to reproduce the neural damage of an ischemic stroke, in which deoxygenation caused by arterial occlusion produces definite and circumscribed neuronal necrosis (22). On the other hand, diffuse lesions resulted in neuronal deactivation in a distributed manner without causing a complete regional activity loss. This approach aimed at reproducing the consequences of diffuse axonal injury and metabolic alterations typical of traumatic brain injuries (TBI) (36) (Fig. 2*B*). Indeed, while post-TBI diffuse axonal injury typically involves white matter tracts and produces widespread network disruptions (37), strokes cause functional impairments that reflect the regional interruption of vascular flow (38).

Representative simulations of lesion experiments are summarized in Fig. 2*A*. To evaluate how these different lesions alter network dynamics beyond behavioral scores, we tracked changes in single neurons’ firing rate, the branching ratio (baseline ${\hat{\sigma }}_{net}$ = 0.9881, near criticality) via a multistep-regression (MR) estimator (39), and the Brunel synchrony index (χ^2^) across all lesion types and severities (Fig. 2*C*).

### Inhibitory Lesions.

At low lesion severity, inhibitory lesions had minimal impact on network task performance. For instance, after a 7% inhibitory lesion (2 neurons), mean score (±SEM) for local lesions was 0.94 (±0.02) and 0.97 (±0.01) for diffuse lesions, with no significant difference between lesion types (*P* = 0.23). At moderate severity, however, performance decreased remarkably for local lesions: A 43% lesion (12 neurons) resulted in a score of 0.84 (±0.03) for local versus 0.95 (±0.01) for diffuse lesions, with a Bonferroni-corrected significant difference (*P* = 0.005, Cohen’s *d* = −1.17). Severe lesions (86%; 24 neurons) reduced performance to chance level, with a mean score of 0.53 (±0.03) for local lesions and 0.59 (±0.03) for diffuse ones. When testing lesions in the larger model of 200 total E/I neurons, the network showed higher resilience and better recovery with the same % of lesions; however, the same spontaneous recovery pattern was observed, with local lesions leading to worse recovery at moderate severity.

In terms of network dynamics, inhibitory lesions showed a nonmonotonic effect (Fig. 2*C*). While mild lesions did not alter the branching ratio, network synchrony declined. Moderate inhibitory loss (64%) elevated the firing rate by (+20.2 ± 2.0) Hz, increased the branching ratio $\Delta {\hat{\sigma }}_{net}$ by (+6.63 ± 0.54) × 10^−3^, and decreased synchrony Δχ^2^ by −0.0094 ± 0.0022, while higher loss (86%) attenuated the rate and $\Delta {\hat{\sigma }}_{net}$ elevation ((+8.3 ± 1.1) Hz and (+3.49 ± 0.24) × 10^−3^).

### Excitatory Lesions.

Similar to inhibitory lesions, minor excitatory lesion simulations had minimal impact on task performance. After a 3% excitatory lesion (3 neurons), mean score (±SEM) was 0.93 (±0.02) for local lesions and 0.97 (±0.01) for diffuse lesions, with no significant difference (*P* = 0.10). However, excitatory lesions showed an earlier onset of significant impairment compared to inhibitory lesions. At 7% severity (8 neurons), local lesions determined substantially reduced performance with a mean score of 0.83 (±0.01) versus 0.94 (±0.02) for diffuse lesions, with a Bonferroni-corrected significant difference (*P* < 0.001, Cohen’s *d* = −2.09). Interestingly, at moderate severity levels (21% lesions; 24 neurons), the recovery pattern reversed compared to inhibitory lesions, with diffuse lesions showing worse performance: 0.71 (±0.02) for diffuse versus 0.80 (±0.02) for local lesions (Bonferroni-corrected significant difference, *P* < 0.001; Cohen’s *d* = 1.61). After more severe lesions (34% and above), both lesion types reduced performance to chance level.

The larger neuronal network demonstrated enhanced resilience to excitatory lesions compared to the standard configuration. Additionally, the recovery after local lesions was consistently worse than for diffuse lesions across medium-large lesion sizes (up to 84%), more similarly to inhibitory-only lesions.

Dynamical characterization after excitatory lesions showed a steady reduction in network activity and the branching ratio, while producing a more synchronized regime (Fig. 2*C*). For instance, a 57% excitatory lesion decreased the firing rate by (−11.0 ± 1.6) Hz, dropped $\Delta {\hat{\sigma }}_{net}$ by (−1.58 ± 0.40) × 10^−3^, and raised the Brunel synchrony index Δχ^2^ by +0.026 ± 0.004.

### Combined Lesions.

Both excitatory and inhibitory lesions were then performed. Mild severity lesions (3%; 3 excitatory + 1 inhibitory neurons) did not impact the model’s recovery, with mean score (±SEM) at 0.94 (±0.02) for local versus 0.95 (±0.01) for diffuse lesions. At moderate severity levels (7%; 8 excitatory + 2 inhibitory neurons), local lesions had a greater impact on recovery than diffuse lesions: 0.78 (±0.02) versus 0.87 (±0.04), with no statistically significant difference. At 21% severity (24 excitatory + 6 inhibitory neurons), the pattern reversed with diffuse lesions showing worse performance: 0.71 (±0.03) for diffuse versus 0.83 (±0.02) for local lesions (*P* = 0.006, Cohen’s *d* = 1.12). At more severe lesion levels (35% and above), both lesion types reduced performance to chance level.

When testing lesions with larger network sizes, similar to the single-population lesions, the model demonstrated a higher resilience. Moreover, the recovery pattern changed analogously to excitatory-only lesions. In fact, at small to moderate lesion sizes, local lesions were significantly more detrimental than the diffuse ones.

Combined lesions, at matched severity, produced a smaller dynamical shift than excitatory-only lesions. For instance, a 57% combined lesion resulted in a smaller synchrony increase (Δχ^2^ +0.004 vs +0.026) and a more moderate drop in firing rate (Δrate −7.3 vs −11.0 Hz) than an equivalent excitatory-only lesion. This indicates that, despite comparable behavioral scores, their underlying dynamical regimes differed.

### Inhibitory Postsynaptic Potentiation.

**Fig. 3.**
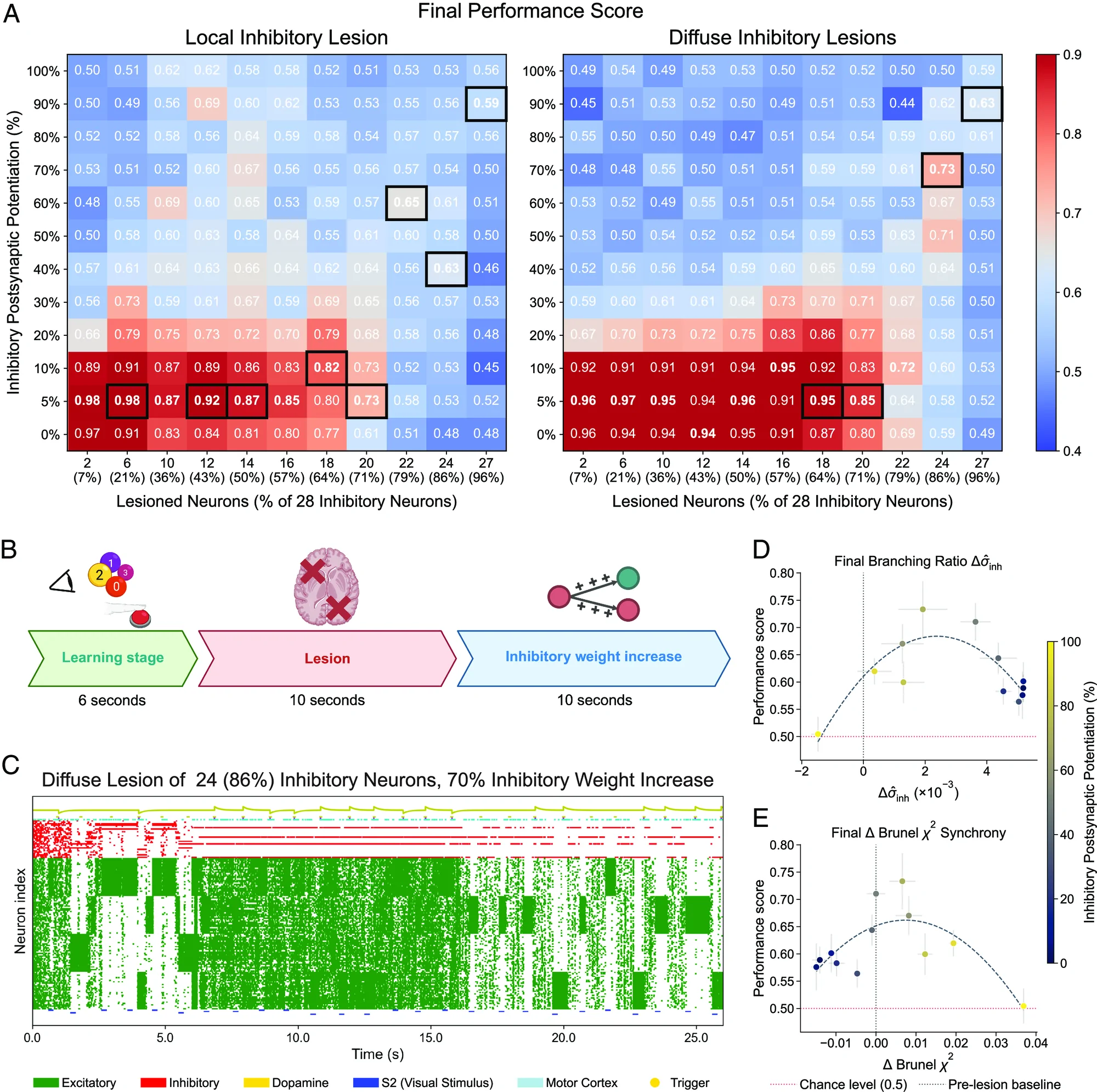
Simulations of GABA_A_ receptor positive allosteric modulation after inhibitory lesions. (*A*) Final score after inhibitory lesions and GABA_A_ receptor PAM simulation. The heatmaps show final performance score (color scale), as the mean across n = 10 independent trials per cell, across lesion size and inhibitory postsynaptic potentiation. Recovery is noticeable in medium and large size lesions at around 5 to 10% inhibitory weight increase (expressed here as inhibitory postsynaptic potentiation) in local lesions, and between 10 to 70% in diffuse lesions. Bold values highlight the highest score within each lesion size, and bordered cells mark best performance when it exceeds the 0% control by ≥0.05. (*B*) GABA_A_ receptor PAM potentiation experiments design. After 6 s of regular learning, a lesion is introduced, followed by 10 s of spontaneous recovery. Inhibitory synaptic weights are then increased to simulate GABAergic medication. (*C*) Recovery. The spike raster plot (temporally aligned with panel *B*) illustrates neuronal hyperactivation following an inhibitory lesion and subsequent restoration of winner-takes-all (WTA) dynamics after PAM potentiation. (*D*) Performance relationship with inhibitory branching ratio ($\Delta {\hat{\sigma }}_{inh}$). Final performance plotted against changes in the branching ratio of the surviving inhibitory population after the illustrative 86% diffuse inhibitory lesion. Points are per-dose means; error bars are ±SEM; the red dotted line marks chance (0.5). The inverted-U trajectory reveals that successful behavioral rescue correlates with precise tuning of remaining inhibitory networks (quadratic vs linear, extra-sum-of-squares F-test; *R*^2^ = 0.713, F(1,9) = 22.29, *P* = 0.001; n = 12 inhibitory potentiation levels). (*E*) Performance relationship with network synchrony (Δχ^2^). As in (*D*), final task performance against changes in the Brunel synchrony index for the same 86% diffuse inhibitory lesion scenario. This confirms that effective inhibitory postsynaptic potentiation relies on the retrieval of a balanced global dynamic (*R*^2^ = 0.603, F(1,9) = 13.33, *P* = 0.005).

The simulated inhibitory postsynaptic potentiation showed a dose-dependent efficacy on recovery after inhibitory neuron lesions (Fig. 3*A*), with Bonferroni-corrected significant improvements observed in the most severe lesion sizes, though recovery in these cases plateaued slightly above chance level. At high lesion severity, inhibitory weight increase demonstrated different therapeutic windows based on lesion type. For diffuse lesions of 24 neurons (86% damage), we observed sub-optimal recovery with 70% inhibitory weight increase, resulting in a final mean score (±SEM) of 0.73 (±0.05), a statistically significant improvement compared to control (*P* = 0.015). Local lesions showed a higher sensitivity to lower inhibitory enhancement compared to diffuse lesions: At 20 neuron lesions (71% damage), 10% inhibitory weight increase resulted in a notable recovery with a mean score of 0.68 (±0.03) and significant improvement (*P* = 0.048) when compared to control condition. Higher GABA_A_ PAM action at smaller lesion sizes generally worsened the model’s performance.

Inhibitory enhancement after only excitatory and combined lesions did not produce substantial recovery after moderate and large lesion sizes (*SI Appendix*, Fig. S1).

To explore the underlying dynamics under the performance rescue in the inhibitory lesion condition, we examined the relationship between performance and network dynamics across simulated GABA_A_ PAM doses in the illustrative 86% diffuse inhibitory lesion (Fig. 3 *D*–*E*). Because PAM directly increases inhibitory postsynaptic weights, branching ratio was measured among the inhibitory population ($\Delta {\hat{\sigma }}_{inh}$), which operated near criticality at baseline (prelesion $\Delta {\hat{\sigma }}_{inh}$ = 0.9947). Recovery followed an inverted-U trajectory against $\Delta {\hat{\sigma }}_{inh}$ (quadratic R^2^ = 0.713, *P* = 0.001) and Δχ^2^ (quadratic R^2^ = 0.603, *P* = 0.005). Peak performance occurred at an optimal vertex shift of +2.38 × 10^−3^ for the branching ratio (significantly above baseline) and a shift consistent with the prelesion level for network synchrony (95% CI including zero). This shows that successful network recovery relies on tuning the network into a precise dynamical window (full statistics reported in *SI Appendix*, Table S2). The firing rate showed a weaker tendency toward the same shape, and network-wide ${\hat{\sigma }}_{net}$ showed no significant inverted U-shape (*SI Appendix*, Table S2). Ultimately, this suggests that GABAergic PAM efficacy relies on sufficient surviving inhibitory activity, whose potentiation can dynamically restore the E/I balance, rescue a critical network state, and reinstate GNW functions.

### Robustness Across E/I ratio and Input Drive.

Finally, we assessed whether our findings depend on specific parameter choices; we replicated the two illustrative inhibitory lesion-intervention scenarios (71% local lesion with 10% inhibitory postsynaptic potentiation, and 86% diffuse lesion with 70% potentiation) across E:I ratios of 70:30 and 90:10, and spontaneous intrinsic activities of 6, 9, and 15 Hz (*SI Appendix*, Table S3). Recovery patterns were preserved: Statistical significance was reached in the diffuse lesion scenario across all tested E:I ratios and at 15 Hz, while local lesions recovered significantly only at the 90:10 E:I ratio. No learning occurred at 6 Hz, in line with Volzhenin et al. (21). The findings hold across spontaneous input rates of 9 to 15 Hz and E:I ratios from 70:30 to 90:10, with 12 Hz and an 80:20 ratio used as biologically plausible operating points for testing GNW architecture and function, supporting trace conditioning learning. This showed that the lesion-GABAergic PAM framework may be relevant beyond computational abstractions, as in biological settings where E/I balance and firing rates naturally vary.

## Discussion

The SNN experiments provided a mechanistic investigation on network dynamics underlying conscious access, examining how lesion patterns, E/I imbalances, and inhibitory modulation may account for the apparent paradox of GABAergic responsiveness in DoC.

Our biologically constrained model incorporates several features that have been previously individually addressed but rarely combined in a single framework. In fact, many computational models of conscious processing primarily simulate neural activity without incorporating temporal dynamics or plasticity rules (40).

The model’s assumptions are based on the Global Neuronal Workspace Theory (GNWT), which posits that conscious processing emerges when information is broadcasted across cortical and subcortical networks (41). The theory proposes that conscious access is set up by an initial ignition (a large-scale activation across the network) that is subsequently maintained by a reverberant network, ensuring its sustenance over time (42).

Accordingly, the model learned the trace conditioning task, achieving optimal performance through stable column-specific activation. This simulated the *ignition* and *reverberation* underlying conscious access, as predicted by the GNWT (42).

### Lesions and Spontaneous Recovery.

When the model was subjected to diffuse and local lesions toward the excitatory population, it reacted differently depending on lesion size and type. After minimal lesions, the model showed effective adaptation through plasticity: Ongoing STDP reorganized the surviving connections, preserving function despite the initial damage, mirroring recovery from realistic minor lesions (43).

At small lesion sizes, local lesions impaired recovery more severely. This divergence is closely related to the model’s columnar architecture and task design. Local excitatory lesions of this size selectively disabled a single column, reducing its surviving neurons below the minimum needed to function and abolishing its contribution to task performance. Conversely, diffuse lesions of the same size spread the damage across multiple columns, allowing room for spontaneous reorganization and recovery.

However, as lesion size increased, this advantage diminished: The growing impact of diffuse damage eventually overtook that of local lesions by simultaneously disrupting multiple columns, whereas local impairment still ensured the performance of the remaining columns. Medium-sized diffuse lesions likely pushed all columns below this critical threshold simultaneously, causing widespread collapse. This interpretation is supported by our experiments with larger networks, in which local lesions remained consistently more detrimental than diffuse lesions across medium lesion sizes. This suggests that the additional neurons provided an adequate buffer against general collapse, in line with explored network compensation mechanisms after neuronal loss (44).

When damage targeted exclusively inhibitory neurons, recovery trajectories differed. After both local and diffuse small lesions, the model gradually regained optimal performance, indicating a higher degree of redundancy within the inhibitory population. As lesion size increased, however, local inhibitory damage undermined the model’s performance more than diffuse lesions. Even with more extensive diffuse lesions, inhibitory activity showed greater resilience, revealing better performance across medium to large lesion sizes compared to excitatory lesions (Fig. 2*A*).

This discrepant behavior reflects the role of inhibitory neurons in biological systems: They exert control over winner-takes-all (WTA) dynamics (45), stabilize the network’s activity (46), but do not directly handle the task-specific demands. In our model, inhibitory circuits are less vulnerable to medium-size diffuse damage; a few evenly distributed inhibitory neurons can preserve this regulatory function.

Combined lesions reproduced the excitatory-only damage pattern, with performance dropping to chance level at similar thresholds as excitatory-only lesions (35%); however, their dynamical signature diverged, revealing a dissociable inhibitory contribution masked by the behavioral outputs.

While clinical studies exploring specific neuronal population loss are limited (47), preclinical evidence in the mouse hippocampus suggests that mature excitatory neurons are more vulnerable than inhibitory interneurons after hypoxic-ischemic damage (48). Although our model did not assign differential susceptibility, inhibitory connectivity persisted better than excitatory after equivalent lesions. This suggests that biological networks may maintain inhibitory control even after mid-sized injuries, consistent with evidence that inhibitory circuits adapt to preserve control after TBIs (49).

Ultimately, high lesion sizes caused anomalous network activity. In our model, the collapse of selective and competitive columnar activation coincided with a shift toward network oversynchronization following excitatory and combined lesions (Fig. 2*C*). This relates, at a different scale, to the pathological between-network hyperconnectivity reported in DoC, where recovery is marked by the restoration of normal anticorrelations (50).

### GABA_A_ Receptor PAMs After Lesions.

Inhibitory synapses hyperpolarize the postsynaptic neurons, reducing their likelihood of generating action potentials (51). We simulated GABAergic modulation by proportionally increasing inhibitory synaptic weights (I → E and I → I), reproducing the enhanced inhibitory postsynaptic potentials (IPSPs) (52).

After local inhibitory lesions (Fig. 3*A*), when 71% of inhibitory neurons were removed, a 10% increase in inhibitory weights restored task performance above chance, within a window of low percentage potentiation. After larger diffuse inhibitory damage (86%), a 70% increase in inhibition partially restored the model performance. Further larger lesions and higher GABAergic enhancements did not restore network function, suggesting a ceiling effect when inhibitory connections are highly compromised. These results support the hypothesis that the benefits of inhibitory postsynaptic potentiation in DoC depend primarily on the lesion’s nature, particularly its pattern and size (53). Whether surviving inhibitory neurons were evenly distributed or localized, potentiating their connections rebalanced global E/I dynamics and facilitated GNW-like ignition and reverberation (42), and their distribution influenced the required dose and the attainable ceiling (Fig. 3*A*). Conversely, excitatory or combined lesions showed no recovery regardless of inhibitory enhancement or lesion size and pattern.

This evidence supports the mesocircuit hypothesis of DoC, which posits that arousal after GABAergic medications in some patients results from restored inhibitory signal, with particular emphasis on the basal ganglia: specifically, a reinstated inhibition of the Globus Pallidus (GP), followed by disinhibition of the thalamus (16). The GP interna (GPi) has been described as the fulcrum of a broader inhibitory restoration (involving GABA_A_ receptors in the frontal cortex and striatum), since it is insensitive to reduced background activity (e.g., during anesthesia) while featuring higher density of zolpidem-binding receptors (54). The same recovery can be interpreted through the criticality framework. Inhibitory lesions shifted the network toward a supercritical-leaning regime, defined here by the elevated branching ratio (Fig. 2*C*). The branching ratio and χ^2^ moved inversely: Inhibitory loss raised σ while reducing synchrony, whereas excitatory loss caused the opposite shift. The intervention then rescued performance by returning ${\hat{\sigma }}_{inh}$ and χ^2^ near, yet offset from, prelesion baseline (Fig. 3 *D*–*E*). This state, observed in responsive combinations, reflects a critical regime: Precise, task-specific activation of neuronal columns is sustained through stable excitatory–inhibitory interactions (55).

Empirical support for these possible mechanisms underlying GABAergic responsiveness in DoC comes primarily from zolpidem studies, which demonstrate the clearest clinical improvement. Cases with basal ganglia involvement (56–59) showing zolpidem responsiveness align with mesocircuit dysfunction, where disrupted striatum-GP signaling appears restored by GABAergic enhancement. Beyond pharmacology, in chronic moderate-to-severe TBI, central lateral thalamic Deep Brain Stimulation (DBS) has been proposed to act by reversing a down-regulated GABAergic signal across frontostriatal circuits (60). Moreover, sedatives and anesthesia may push neural networks toward subcriticality (5). The observed responsiveness to zolpidem across different etiologies, with marked slow-wave activity in common (61–68), is in line with the critical brain hypothesis, as inhibitory medications may shift supercritical networks back toward critical dynamics (17), even in absence of a common structural brain involvement. Dynamical consequences of E/I imbalance are measurable in vivo. The branching parameter itself can be extracted from EEG, as well as the aperiodic component of the signal, whose spectral slope can indicate cortical E/I balance. Notably, the preanesthetic slope conditions the magnitude of the response to a GABA_A_ PAM like propofol (69), a baseline dependence consistent with that observed in our simulations. Additionally, some responsive patients had primary occipital lesions (70, 71). Since GABA receptor density is highest in the occipital lobe (72, 73), it is presumable that lesions in receptor-dense regions produce inhibitory deficits responsive to pharmacological inhibitory postsynaptic potentiation. To our knowledge, this hypothesis has not yet been tested and lacks direct empirical support. However, it has been highlighted the possible role of cortical and striatal inhibitory neural networks in reinstating inhibitory drive (62). This could be explored in future research using existent brain maps of receptor densities (74) or by characterizing GABA_A_ receptor availability with [^11^C]-flumazenil positron emission tomography (FMZ-PET). For instance, longitudinal tracking after TBI showed both a mesocircuit signature (persistent frontal reductions alongside increased pallidal binding) and that the recovery of executive attention correlated with widespread cortico-thalamic increases in receptor availability (75). Similarly, anti-NMDA receptor encephalitis affects inhibitory neurons at key nodes, including the hippocampus and prefrontal cortex, with a reduction of inhibitory tone (76) that can be restored after zolpidem administration (77).

The distinct pharmacodynamic profiles of zolpidem (alpha-1 selective GABA_A_ PAM) (78), propofol (nonselective GABA_A_ PAM) (79), and sodium oxybate and baclofen (GABA_B_ agonists) (80, 81) may confer differential advantages depending on the specific mechanism of E/I imbalance involved; for instance, reported baclofen responses involve intrathecal administration and emerge gradually on slower timescales (15). More broadly, DoC interventions such as dopaminergic and glutamatergic agents (e.g., amantadine, apomorphine) and neuromodulation techniques [e.g., central-thalamic DBS, repetitive Transcranial Magnetic Stimulation (TMS), and transcranial direct current stimulation (tDCS)] impact network-level dynamics and have been described under the same mesocircuit and E/I imbalance framework (82). Tailoring interventions to a patient’s specific circuit dysfunction may significantly improve clinical outcomes.

Several methodological constraints must be considered when interpreting our findings and their translation to clinical settings. We lesioned excitatory and inhibitory populations separately to isolate each population’s contribution to network dynamics, similarly to empirical studies with cell-specific perturbations [e.g., optogenetic silencing (83)], without presupposing that clinical injuries are population-specific; therefore, inhibitory-only lesions simulate a functional rather than a purely numerical imbalance. Moreover, anatomically mixed damage can produce a functional inhibitory deficit: For instance, energy-demanding parvalbumin-positive interneurons are more vulnerable to moderate hypoxia (79). Additionally, the model features the GNW architecture and not anatomically specified regions, and operates at a relatively small scale. The incorporation of subcortical structures, such as the central thalamus, whose excitatory–inhibitory interplay has been identified as a substrate for DoC recovery outcomes (84), will enhance biological accuracy.

Computational outputs should not be interpreted as reproductions of DoC’s clinical hallmarks but as tools to test mechanistic hypotheses. For instance, the task performance indexes the ignition and reverberation of a computational signal; therefore, it should not be conflated with multidimensional arousal and awareness scales such as the CRS-R or SECONDs (85). The 12 Hz intrinsic spontaneous activity is an input drive that sustains a learning-capable baseline and does not emulate resting-state spectral signatures of DoC (16). Other constraints, such as synchronous synaptic weight updates and the lack of molecular distinction of different GABAergic medications, are limitations that will be addressed in future work that will extend the framework (e.g., by incorporating different neurotransmitters, interneuron subtypes, and subcortical structures with heterogeneous receptor distributions) to increase clinical relevance.

### Conclusions: A Testable Unifying Hypothesis.

Our computational findings support a unifying framework to explain the apparent paradox of the therapeutic effects of GABAergic compounds in DoC across diverse neurological damage. We propose that insufficient inhibition within E/I dynamics is the common pathway in responsive cases. This multilevel investigation (covering molecular modulation, circuit reorganization through plasticity, network dynamics, and conscious access) highlights the complementarity of the mesocircuit and criticality hypotheses: the former specifies where, anatomically, the inhibitory deficit lies (whether subcortical in striatal-GP signaling or cortical regions in receptor-dense or autoimmune-affected circuits) and where its restoration may be leveraged, while the latter specifies the dynamical regime that this rebalance corrects, regardless of structural integrity. Consistently, focal lesions that interrupt pathologically sustained activity can themselves restore function, as in the Sprague effect and related paradoxical facilitation phenomena (86, 87), suggesting that recovery hinges on rebalancing disrupted network dynamics.

In conclusion, predicting responsiveness to GABAergic medications may require combining neuroimaging techniques with spatial resolution (such as FMZ-PET (75) to identify mesocircuit or GABA receptor-dense region involvement) and temporal resolution (e.g., EEG-based criticality metrics like the branching parameter and the Hurst exponent (5) to detect supercritical states). These markers could be complemented with ^18^F-FDG-PET for differentiating levels of awareness (88), and with the perturbational complexity index (PCI) from TMS-EEG, a measure of integrated and differentiated activity (89) that could map onto the criticality transitions our SNN predicts (Table 1). Validating this model in future studies could establish it as a precision medicine tool, offering prospective analytical approaches for these challenging neurological conditions.

**Table 1.** Translational map from network disruption to GABAergic responsiveness

Network disruption	Model’s dynamic signature	GABAergic modulation	Potential clinical assessment
Predominant inhibitory disruption	Increased branching ratio	Responsive to GABAergic modulation	EEG-based branching ratio, aperiodic spectral slope; FMZ-PET/FDG-PET
Predominant excitatory disruption	Lowered branching ratio	Nonresponsive to GABAergic modulation	

Summary of the model’s dissociation between inhibitory and excitatory-predominant disruption, their dynamical signatures, predicted GABAergic responsiveness, and candidate clinical readouts.

EEG, electroencephalography; FMZ-PET, [^11^C]-flumazenil positron emission tomography; FDG-PET, ^18^F-fluorodeoxyglucose positron emission tomography.

## Materials and Methods

### Model’s Characteristics.

The code is available on https://github.com/antoinecautru/Epigenesis_pytorch_miniBinsnet (32). Our SNN was built in Python 3.9.2, leveraging the PyTorch library for neural network operations. The code architecture took inspiration from two main sources. BindsNET, a machine learning-oriented SNN (33), mainly influenced the fundamental structure, while the hierarchical network architecture was modeled after the sensorimotor, cognitive, and conscious framework described by Volzhenin et al. (21). A time step parameter *dt* allowed the synchronous updates of network parameters through different batches, handled by a reduction function. Our model incorporated an S2 input layer that mimics visual stimulus processing, a cognitive and conscious level featuring both excitatory and inhibitory populations, and a motor cortex (MC) component that simulates the behavioral responses in a trace conditioning task.

### Spatialization.

Inspired by cortical columns organization (90), the GNW layer was partitioned into distinct columns, each receiving visual input from S2. Excitatory connections were limited to neurons within the same column, while inhibitory neurons were configured to project connections globally, across all columns. This design aimed to reflect the dynamics of lateral inhibition, where interneurons suppress the activity of nearby columns (91). This different connectivity pattern aimed at recreating the biological role of excitatory neurons with local, process-specific features, while inhibitory interneurons provide global regulation and support for competition between columns, resulting in winner-takes-all (WTA) dynamics essential for task execution.

### Lesions.

This spatial organization enabled the implementation of two distinct lesion types to investigate network spontaneous recovery and DoC: 1. diffuse lesions, where a specified number of neurons were randomly selected within the neuronal workspace surface and deactivated; 2. local lesions, which specifically leveraged the spatial organization by first randomly selecting a center, and then deactivating its nearest neurons. Both lesion types could be applied to either excitatory or inhibitory populations. Lesioned neurons were permanently silenced by keeping their membrane voltages at resting potential, removing them from computational operations while maintaining the network architecture.

### GABA_A_ Receptor PAMs.

To simulate the pharmacological GABA_A_ PAM inhibitory effect, we increased the maximum synaptic weight of all inhibitory connections by a predefined percentage. This effect was applied exclusively to connections originating from inhibitory neurons, targeting both inhibitory and excitatory postsynaptic populations.

### Trace Conditioning Task and Scoring System.

The trace conditioning task involved the sequential presentation of visual stimuli (digits from 0 to 3), which served as the conditioned stimuli (CS). Each episode followed a fixed temporal structure lasting 1,000 ms: Visual stimulus lasted 150 ms, with spikes delivered at 3 ms intervals. This was followed by a stimulus-free trace interval of 150 to 250 ms, during which no stimulus was delivered. The episode concluded with a 50 ms trigger, representing the unconditioned stimulus (US), and a relaxation period before the onset of the next episode.

The model had to provide a motor reaction during the US when the visual stimulus was an even number. The performance was quantified through a scoring system that evaluated a dopaminergic signal fired after the provided response. The calculation of the score assessed four metrics: true positive (dopamine release when right answer), false positive (dopamine release when incorrect answer), false negative (no dopamine release when expected), and true negative (no dopamine when required). The final performance score was calculated as the proportion of the correct responses:

$$score=1-\frac{N_{\mathrm{wrong}}+N_{\mathrm{missed}}}{N_{\mathrm{total}}},$$

where N_wrong_ represented the inappropriate dopaminergic responses and N_missed_ the lack of dopaminergic response when required. The task performance ranged from 0 (worst performance) to 1 (optimal performance), with 0.5 representing chance level.

Scores across trials were measured with mean score and SEM.

### Lesions and Inhibitory Postsynaptic Potentiation Experiments.

The model featured a total of 149 neurons: four at the S2 level, 144 at the conscious level (organized into four columns of 36 neurons each), and one representing the motor output. We maintained biologically plausible features from the model developed by Volzhenin et al., including an 80:20 E:I ratio and a spontaneous intrinsic activity of 12 Hz (21). Spontaneous intrinsic activity was implemented as independent Poisson-distributed external input, delivered to each excitatory and inhibitory neuron. It provided the necessary drive that sustained baseline network activity and trace conditioning learning.

To assess the effects of lesions, the model was first trained on the trace conditioning task for 6 s (equivalent to six episodes). After initial learning, we introduced either localized or diffuse lesions of varying sizes, followed by a 10-s spontaneous recovery period. For each lesion condition, 10 independent trials were run.

We evaluated three lesion types: inhibitory-only, excitatory-only, and combined inhibitory and excitatory lesions. The lesion sizes were as follows:

- Inhibitory lesions: Out of 28 inhibitory neurons, we deactivated 7% (2), 21% (6), 36% (10), 43% (12), 50% (14), 57% (16), 64% (18), 71% (20), 79% (22), and 86% (24).
- Excitatory lesions: Out of 116 excitatory neurons, we deactivated 3% (3), 7% (8), 10% (12), 14% (16), 17% (20), 21% (24), 34% (40), 57% (66), 71% (82), and 84% (98).
- Combined lesions: We inflicted same-percentage lesions across both populations as follows: 3% (3 excitatory + 1 inhibitory), 7% (8 + 2), 10% (12 + 3), 14% (16 + 4), 17% (20 + 5), 21% (24 + 6), 35% (40 + 10), 57% (66 + 16), 71% (82 + 20), and 85% (98 + 24).

To assess whether recovery patterns were influenced by network size, we scaled the conscious-level population up to 200 neurons and tested analogous lesion combinations.

Because trials differed only in lesion topology, we conducted statistical analysis using paired *t* tests to compare local versus diffuse lesion types across severities, evaluating task performance at the end of the simulations. Effect sizes were calculated using Cohen’s *d*. To account for multiple comparisons, we applied Bonferroni correction (for each lesion type; α = 0.05/10 = 0.005). Reported *P*-values are uncorrected, with Bonferroni significance specified where applicable.

In the inhibitory postsynaptic potentiation experiments, the first part of the simulations mirrored the experiments with lesions, with the inhibitory 96% lesion condition added here, this time allowing 10 additional seconds with inhibitory connection weights increased to 0%, 5%, 10%, 20%, 30%, 40%, 50%, 60%, 70%, 80%, 90%, and 100% (Fig. 3*B*). Ten independent trials were performed for inhibitory lesion combinations, 5 for excitatory and combined lesions. Statistical analyses were conducted using the paired *t* test to compare each condition against its corresponding 0% control for the same lesion type and size. Effect sizes were quantified using Cohen’s d. To account for multiple comparisons, Bonferroni correction was applied separately within each lesion size (α = 0.05/11 = 0.0045), as each lesion severity represented a distinct biological condition with potentially different dose–response relationships. Again, reported *P*-values are uncorrected, and Bonferroni significance is specified where applicable.

### Dynamical Characterization.

Network dynamics were quantified over three windows (prelesion, postlesion, post-PAM) from spike trains binned at 1 ms. We computed the neurons’ firing rate, the Brunel synchrony index χ2 (ratio between population activity variance and single-neuron variance) (92), and the branching ratio σ as an estimate of network criticality, obtained with the multistep-regression (MR) estimator (39). Metrics were computed separately for the excitatory, inhibitory, and pooled (net) population and averaged across trials. Postlesion changes were reported relative to the mean of the prelesion window.

We then related post-PAM performance to each dynamical metrics across PAM percentages in the illustrative 86% diffuse inhibitory lesion. We fit performance against the metrics’ Δ with linear and quadratic polynomials, tested the quadratic fit with an extra-sum-of-squares F-test, and estimated the inverted-U vertex with its 95% bootstrap CI and a leave-one-dose-out range. We report the inhibitory-population branching ratio (σinh) and χ2 in the main text as the most mechanistically relevant readouts; σnet, the Fano-like synchrony index, and the post-PAM firing rate, either less sensitive or collinear with the reported metrics, are provided in the *SI Appendix*.

### Robustness Across E/I Ratio and Input Drive.

To verify that our results did not depend on specific parameter choices, we replicated the two illustrative inhibitory lesion-intervention scenarios (71% local lesion with 10% inhibitory postsynaptic potentiation, and 86% diffuse lesion with 70% potentiation) while independently varying the E:I ratio (70:30 and 90:10) and the spontaneous intrinsic activity (6, 9, and 15 Hz). As the inhibitory population size differed across ratios, lesion sizes were bracketed around the target damage with the nearest neuron counts. As in the main experiments, inhibitory weights were increased from 0 to 100%, 10 independent trials were run per condition, and each inhibitory potentiation was compared against its 0% control using the paired *t* test with Bonferroni correction (α = 0.05/11).

## Supplementary Material

Appendix 01 (PDF)

## Data Availability

Code data have been deposited in https://github.com/antoinecautru/Epigenesis_pytorch_miniBinsnet (32).
