# Supplementary material for: Recovery from disorders of consciousness: Lesions and GABAergic modulation in a biologically inspired spiking neural network
Source: Proc Natl Acad Sci U S A. 2026 Sep 22;123(39):e2530522123. doi: 10.1073/pnas.2530522123 (PMC13624685; doi:10.1073/pnas.2530522123)
Supplement: Supplementary file 1 [file pnas.2530522123.sapp.pdf]

PNAS

Supporting Information for
Recovery from Disorders of Consciousness: Lesions and GABAergic Modulation in a Biologically-Inspired Spiking Neural Network

Francesco Achilli, Antoine Cautru, Jean-Pierre Changeux*, Guillaume Dumas*

*Corresponding authors: Jean-Pierre Changeux, Guillaume Dumas
Email: changeux@noos.fr, guillaume.dumas@umontreal.ca

This PDF file includes:

Supporting text
Tables S1 to S3
Figure S1
SI References

Supporting Information Text

Batch Processing

Although batch processing is primarily a computational technique employed to reduce simulation time and accelerate iteration speed (1), it can be conceptually aligned with the brain's parallel processing capabilities. In the nervous system, several signals are transmitted and processed in parallel. For instance, the brain's visual system processes information simultaneously through parallel pathways (2), and a similar configuration has been identified in the cortico-cerebellar connectivity (3). Therefore, while not mimicking biological complexities, the use of batch processing can be seen as a practical abstraction of these parallel mechanisms. We aimed to balance computational efficiency with biological plausibility, accelerating the simulations without diverging from the neurobiologically-inspired principles offered in Volzhenin et al. (4).

If the biological plausibility remains debatable, we observed that running identical networks in parallel (shared weights, independent membrane potentials), and averaging the Spike-timing-dependent plasticity (STDP) update of each timestep across the batch of networks, would allow to obtain similar learning curves, the only difference being (i) increased reproducibility, (ii) faster learning (the actual learning time is the original one divided by the batch size) and (iii) increasing instability with growing batch size. We hypothesize that this last point is the main reason why SNN with STDP learning and batch processing are not commonly used.

The batch processing takes the following form. We define a batch of B independent networks, each containing N neurons. All networks share the same set of synaptic weights but evolve independently in terms of membrane potentials and inputs.

1. Initialization All networks are initialized with identical weights and resting membrane potentials:

$$W_{i\to j}^{(b)}(0) = \widehat{W}_{i\to j}(0), \quad V_i^{(b)}(0) = V_{\text{rest}}$$

For all $b < B$, $i, j < N$.

2. Independent Dynamics. Each network *b* receives independent stimulation (background Poisson spikes and external input). The membrane potentials $\mathrm{V}_{\mathrm{i}}^{(\mathrm{b})}(\mathrm{t})$ evolve independently according to the neuron model, while the weights remain shared.

3. Per-Network STDP Update. At each timestep *t*, the spiking activity of network *b* defines a theoretical STDP update:

$$u_{i\to j}^{(b)}(t) \equiv \Delta W_{i\to j}^{(b)}(t) = \mathcal{F}\left(S^{(b)}(t), \left(S^{(b)}(\tau)\right)_{\tau<t}\right),$$

where $\mathcal{F}$ is the STDP rule, depending on present and past spiking activity, but not on the weights.

4. Batch Averaging. The updates are averaged across the batch to form a unique update:

$$\boldsymbol{\widehat{U}_{i\to j}(t) = \frac{1}{B}\sum_{b=1}^{B} u_{i\to j}^{(b)}(t)}.$$

5. Optimizer Transformation. The averaged update is then transformed elementwise as:

$$\Delta W_{i\to j}^{\text{opt}}(t) = \widehat{U}_{i\to j}(t) \cdot \mathrm{f}\left(\left(W_{p\to q}(t)\right)_{p,q<N}\right)_{i,j},$$

where f is a function acting on the full weight matrix W. The function is described later in the document.

6. Weight Update (shared across all networks). Finally, the weights of all networks are updated identically:

$$W_{i\to j}^{(b)}(t+\Delta t) = W_{i\to j}^{(b)}(t) + \Delta W_{i\to j}^{opt}(t), \qquad \forall\, b < B.$$

Optimizer for Synaptic Weight Stabilization

To stabilize the evolution of synaptic weights during STDP learning through batch processing, we incorporated an optimizer that normalizes the evolution of synaptic weights based on the connections already established. Specifically, the weight update for a synapse from presynaptic neuron *i* to postsynaptic neuron *j* is regulated according to:

$$\Delta W_{i\to j}^{\text{normalized}} = \Delta W_{i\to j} \cdot \min\left(\alpha \cdot \frac{N_{\text{in}}^{j} + N_{\text{out}}^{i}}{\sum_k \frac{|W_{k\to j}|}{W_{\max}} + \sum_l \frac{|W_{i\to l}|}{W_{\max}} + \epsilon}, 1 \right) \cdot \left(1 - \frac{|W_{i\to j}|}{W_{\max}}\right)^{\beta},$$

where N_{in}^{j} is the number of input connections to neuron *j*, N_{out}^{i} is the number of output connections to neuron *i*; α is a scale factor modulating the normalization strength; β is a power factor that prevents excessive learning rates for synapses already established. This stabilization strategy is implemented according to two biological considerations: (i) limited spatial capacity of dendrites and axons to form and maintain functional connections (5, 6), and (ii) the metabolic expenses associated with maintaining synapses, which enforce natural limits on the total number of connections per neuron (7).

Dynamic Threshold

Differently from other neuron populations in our model, excitatory neurons featured a dynamic firing threshold, inspired by activity-dependent threshold plasticity. This mechanism reflects the experimental observations where persistently activated neurons become increasingly excitable over time (8). The dynamic threshold is modulated according to the neuron's recent firing history:

$$\frac{d\theta_{dynamic}(t)}{dt} = \frac{A_{past}(t) + \theta_{rest} - \theta_{dynamic}(t)}{\tau_{thresh}}$$

$$\frac{dA_{past}(t)}{dt} = \delta_{activity} \cdot S(t) - \frac{A_{past}(t)}{\tau_{trace}}$$

Here, $A_{past}(t)$ represents the neuron's recent activity trace; θ_{rest} is the baseline resting threshold potential; τ_{thresh} is the time constant controlling threshold adaptation; $\delta_{activity}$ is the activity-dependent increment factor; *S(t)* is the spike output at time *t*; τ_{trace} sets the decay rate of the activity trace.
This calculation established a positive feedback loop that increased the responsiveness of frequently active neurons. Such dynamics is thought to improve homeostatic regulation and facilitate the emergence of neuronal *attractors* through enhanced excitability (9).

Excitatory to Motor Cortex Connections

Unlike the Volzhenin et al. model (4), which implemented a dopamine-modulated STDP for connections between excitatory neurons in the GNW and the MC, we fixed the strength of these latter synaptic weights. This choice was motivated by our specific research focus on GNW adaptation to lesions and therapeutic interventions, and maintaining consistent motor output helped in isolating the effects of network disruptions.

Implementation	Batch size	Final score (mean ± SD)	CV	Sim. time (s)	t(≥0.8) (s)
Brian2	1	0.772 ± 0.033	0.043	250	100
PyTorch + optimizer	1	0.936 ± 0.027	0.029	250	100
PyTorch + optimizer	5	0.919 ± 0.029	0.032	50	30
PyTorch + optimizer	10	0.963 ± 0.030	0.031	25	10
PyTorch + optimizer	20	0.955 ± 0.036	0.037	12.5	7.5
PyTorch + optimizer	50	0.967 ± 0.030	0.031	5	3
PyTorch, no optimizer	1	0.776 ± 0.261	0.337	250	100
PyTorch, no optimizer	5	0.693 ± 0.200	0.288	50	NA
PyTorch, no optimizer	10	0.529 ± 0.095	0.180	25	NA
PyTorch, no optimizer	20	0.498 ± 0.031	0.063	12.5	NA
PyTorch, no optimizer	50	0.515 ± 0.056	0.108	5	NA

Table S1. Comparison of Brian2 and PyTorch implementations of the spiking neural network on the trace-conditioning task. Five independent trials per condition; CV is the coefficient of variation; Sim. time is the total simulated time, which decreases with batch size because identical networks are run in parallel; t(≥0.8) is the amount of simulated time required reaching a score of 0.8. NA (Not Applicable) indicates that a score of 0.8 was never reached within the simulated window.

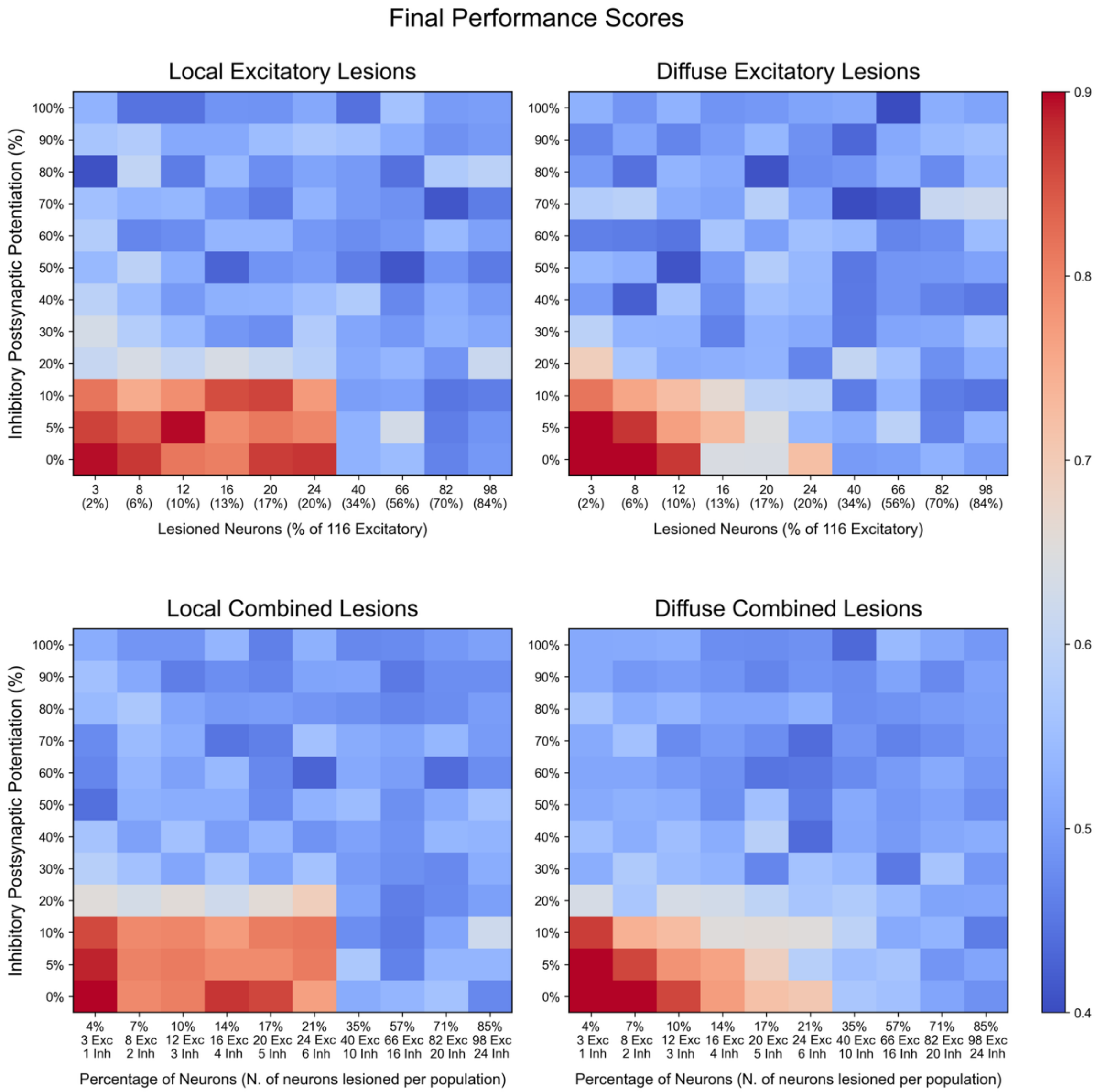

Figure S1. Final score after excitatory (top) and combined (bottom) lesions and $GABA_A$ receptor PAM simulation. In contrast to inhibitory lesions, inhibitory postsynaptic potentiation did not rescue performance or provide substantial recovery.

Statistic	$\Delta\hat{\sigma}_{inh}$(×10^{-3})	Δχ^2 (Brunel)	$\Delta\hat{\sigma}_{net}$ (×10^{-3})	Δ Fano-like synchrony	Δ Firing rate (Hz)
**Pre-lesion baseline**	0.9947 (raw $\hat{\sigma}$)	0.0575	0.9881 (raw $\hat{\sigma}$)	7.5422	36.32 Hz
**R^2, linear fit**	0.001	0.016	0.505	0.013	0.007
**R^2, quadratic fit**	0.713	0.603	0.508	0.612	0.354
**Quadratic vs. linear (F-test)**	$F(1,9) = 22.29$, $p = 1.1\times10^{-3}$	$F(1,9) = 13.33$, $p = 5.3\times10^{-3}$	$F(1,9) = 0.05$, $p = 0.83$	$F(1,9) = 13.91$, $p = 4.7\times10^{-3}$	$F(1,9) = 4.84$, $p = 5.5\times10^{-2}$
**Vertex (optimal Δ)**	+2.38	+0.0074	NA	+0.997	−1.97
**Vertex, 95% bootstrap CI**	[+2.10, +3.01]	[−0.0106, +0.0110]	NA	[−1.49, +1.51]	[−20.1, +15.5]
**Vertex, leave-one-dose-out range**	[+2.28, +2.63]	[+0.0053, +0.0082]	NA	[+0.726, +1.108]	[−11.1, −1.35]

Table S2. Inverted-U fit statistics for the PAM dose-response in the illustrative 86% diffuse inhibitory lesion for all five dynamical metrics ($n = 12$ doses per metric). Performance was fit against each metric's change (Δ) across PAM doses; the F-test compared the quadratic with the linear fit; the vertex is the Δ at peak performance; the 95% CI (2000 bootstrap resamples) and leave-one-dose-out range indexed its stability. $\Delta\hat{\sigma}_{inh}$ and $\Delta\hat{\sigma}_{net}$ are in ×10^{-3}; baselines are in raw branching-ratio units. Fano-like synchrony is collinear with χ^2 (Pearson $r = 1.00$ across doses). NA, no significant quadratic term, hence no vertex defined.

Condition	Lesion	Inh. lesion (%)	Inh. post. pot. (%)	Control score	Post-intervention score	Δ score	p (raw)	p (Bonf.)
**E:I 70:30**	diffuse	95	50	0.465	0.713	+0.248	< 0.001	**< 0.001**
**E:I 70:30**	local	80	20	0.601	0.652	+0.051	0.326	1.000
**E:I 90:10**	diffuse	83	60	0.508	0.678	+0.170	< 0.001	**0.003**
**E:I 90:10**	local	67	80	0.552	0.662	+0.110	< 0.001	**0.007**
**6 Hz**	*No learning*							
**9 Hz**	diffuse	86	30	0.606	0.686	+0.080	0.153	1.000
**9 Hz**	local	71	5	0.675	0.720	+0.044	0.464	1.000
**15 Hz**	diffuse	86	100	0.441	0.682	+0.241	0.004	**0.039**
**15 Hz**	local	71	30	0.606	0.724	+0.118	0.138	1.000

Table S3. Robustness of inhibitory lesion-GABAergic modulation recovery. Two illustrative scenarios (71% local, 86% diffuse inhibitory lesion; lesion sizes bracketed to nearest neuron count) replicated across E:I ratio (70:30, 90:10; intrinsic activity 12 Hz) and intrinsic activity (6, 9, 15 Hz; E:I 80:20). Inhibitory postsynaptic potentiation (Inh. post. pot.) swept 0-100%; the dose with the largest gain over its 0% control is reported (mean of 10 trials). p (Bonf.): paired t-test, Bonferroni-corrected for the 11 doses tested (significance at corrected p≤0.05).

SI References

1. D. J. Saunders, C. Sigrist, K. Chaney, R. Kozma, H. T. Siegelmann, Minibatch Processing for Speed-up and Scalability of Spiking Neural Network Simulation in *2020 International Joint Conference on Neural Networks (IJCNN)*, (2020), pp. 1–8.
2. Y. Shigihara, S. Zeki, Parallel processing in the brain's visual form system: an fMRI study. *Front. Hum. Neurosci.* **8**, 506 (2014).
3. E. J. Müller, *et al.*, Parallel processing relies on a distributed, low-dimensional cortico-cerebellar architecture. *Netw. Neurosci.* **7**, 844–863 (2023).
4. K. Volzhenin, J.-P. Changeux, G. Dumas, Multilevel development of cognitive abilities in an artificial neural network. *Proc. Natl. Acad. Sci.* **119**, e2201304119 (2022).
5. I. Simone Jones, K. Paul Kording, Do biological constraints impair dendritic computation? *Neuroscience* **489**, 262–274 (2022).
6. K. Takagi, Energy constraints on brain network formation. *Sci. Rep.* **11**, 11745 (2021).
7. Y. Yuan, H. Huo, T. Fang, Effects of Metabolic Energy on Synaptic Transmission and Dendritic Integration in Pyramidal Neurons. *Front. Comput. Neurosci.* **12** (2018).
8. K. Ganguly, M. Poo, Activity-Dependent Neural Plasticity from Bench to Bedside. *Neuron* **80**, 729–741 (2013).
9. C. Brennan, A. Proekt, Attractor dynamics with activity-dependent plasticity capture human working memory across time scales. *Commun. Psychol.* **1**, 28 (2023).
